# Remarks from the Editor-in-Chief

**DOI:** 10.1017/psy.2026.10098

**Published:** 2026-03-09

**Authors:** Sandip Sinharay

**Affiliations:** ETS Research Institute Email: ssinharay@ets.org

Dear *Psychometrika* Readers,

Welcome to the first Psychometrika issue of 2026. The call to submit a proposal to IMPS2026 to be held in Seoul, South Korea just ended. I am hoping that many of you submitted proposals to the conference and will be there.

This issue begins with 12 “Theory and Methods” section articles. A trio of articles on item response theory set the tone of this issue. In the first of these, Sainan Xu, Jing Lu, and Jiwei Zhang propose a recursive stochastic algorithm named truncated average stochastic Newton algorithm for the efficient online parameter estimation within the item response theory framework. The second, by Joakim Wallmark and Marie Wiberg, introduces the “bit scale,” a novel metric transformation for unidimensional item response theory scores derived from fundamental principles of information theory: surprisal and entropy. In the third, Daniel Morillo-Cuadrado and Mario Luzardo-Verde generalize the multidimensional discrimination and difficulty parameters in the multidimensional two-parameter logistic model to account for nonidentity latent covariances and negatively keyed items. Two articles on differential item functioning follow. In the first of these, He Ren, Weicong Lyu, Chun Wang, and Gongjun Xu suggest multilevel random item effects models for detecting intersectional differential item functioning. The second, by Ling Chen, Susu Zhang, and Jingchen Liu, proposes a novel method within the framework of generalized linear models for leveraging process data to reduce and understand differential item functioning. Then come three articles on cognitive diagnostic models. In the first of these, Yuqi Gu establishes a new identifiability theory for the Q-matrix used in cognitive diagnostic models; in the second, Jia Liu and Yuqi Gu introduce a new class of models—exploratory DeepCDMs; the third, by Chia-Yi Chiu, Hans Friedrich Köhn, and Yu Wang, derives theoretical foundation of the concepts of proper and plausible multiple choice items for data conforming to a cognitive diagnostic model. The ninth “Theory and Methods” section article of this issue, by Matthias Kloft, Björn Siepe, and Daniel Heck, introduces the interval consensus mode, a novel extension of cultural consensus theory designed to estimate consensus intervals from continuous bounded interval responses. In the tenth article, Daniel Suen and Yen-Chi Chen propose a mixture of binomial experts model for handling neuropsychological test score data that may involve a large degree of missingness. The eleventh article, by Max Welz, Patrick Mair, and Andreas Alfons, proposes a novel estimator that is designed to be robust against partial misspecification of the polychoric correlation model. In the last “Theory and Methods” section article of this issue, Michael Fauss, Xiang Liu, Chen Li, Ikkyu Choi, and H. Vincent Poor investigate the problem of automatically flagging test takers who exhibit atypical responses or behaviors for further review by human experts and develop a selection policy that maximizes the expected number of test takers correctly identified as warranting additional scrutiny while maintaining a manageable volume of reviews per test administration.

This issue then includes three “Application and Case Studies” section articles. In the first, Xiaojing Wang, Abhisek Saha, and Dipak Dey suggest a new class of state space models that jointly handle response times and time series of dichotomous item responses. In the second, Dylan Molenaar and Minjeong Jeon propose a latent space item response models based on two variants of regularized joint maximum likelihood estimation: penalized and constrained. The third, by Andrea Brancaccio, Deborade Chiusole, Ottavia Epifania, Pasquale Anselmi, Matilde Spinoso, Noemi Mazzoni, Alice Bacherini, Matteo Orsoni, Sara Giovagnoli, Irene Pierluigi, Mariagrazia Benassi, Giulia Balboni, and Luca Stefanutti, focuses on models for the assessment of planning skills.

This Psychometrika issue ends with a review written by Yustus Dwi Putera Sepverson Babys of the 2024 book “Principles of Psychological Assessment with Applied Examples in R” authored by Isaac T. Petersen.

Hope you enjoy the issue.

